# Optical and Electrical Characterization of Visible Parylene Films

**DOI:** 10.3390/ma15196717

**Published:** 2022-09-27

**Authors:** Ye-Seul Lee, Ji-Hyeon Yoon, Akeem Raji, Seung-Yo Baek, Yoonseuk Choi, Jonghee Lee, Akpeko Gasonoo, Jae-Hyun Lee

**Affiliations:** 1 Department of Creative Convergence Engineering, Hanbat National University, 125 Dongseo-daero, Daejeon 34158, Korea; 2 Department of Electronic Engineering, Hanbat National University, 125 Dongseo-daero, Daejeon 34158, Korea; 3 Research Institute of Printed Electronics & 3D Printing, Hanbat National University, 125 Dongseo-daero, Daejeon 34158, Korea

**Keywords:** parylene-C, insulation coating, chemical vapor deposition, visible parylene

## Abstract

Poly-dichloro-para-xylylene (parylene-C) film is formed through a chemical vapor deposition process, where monomeric gases are polymerized on the target surface at room temperature and are used as transparent insulating coating films. The thin parylene-C films exhibit uniform conformal layers even when deposited on substrates or surfaces with fine cracks, structures, and bumps. However, the film is highly transparent in the visible range (transmittance > 90%); thus, it is difficult to visually identify, inspect the coating process and check for any defects when used as an insulation film. Some reports have demonstrated the deposition of visible (hazy) parylene films through the control of the vaporization or pyrolysis of the parylene-C powder and sublimed dimers, respectively. Even though these films have been applied as device substrates and light extraction layers in organic light-emitting diodes (OLEDs), their optical and electrical characteristics have not been extensively explored, especially for their applications as insulation coatings. In this study, the characteristics of visible parylene films produced by tuning the ratio of dimer to monomer gases via the adjustments of the pyrolysis temperature are analyzed with electrical and optical methods. Parylene-C films deposited within the pyrolysis temperature of 400–700 °C exhibited a haze range of 10–90%. A relative reflectance of 18.8% at 550 nm of the visible light region was achieved in the visible parylene film deposited with a pyrolysis temperature of 400 °C. Resistivity in the order of 10^10^ Ω cm was achieved for the visible parylene films measured with the transmission line measurement (TLM) method. The films can be applied in advanced insulation coatings for various optical systems and electronic devices.

## 1. Introduction

Poly-para-xylylene (parylene) is a polymer that forms a high-quality thin film of uniform thickness, even on surfaces with fine cracks or complex structures [1]. Parylene film has chemical and electrical resistance, so even when it is coated on the surface of precise electronic components, it does not affect the electrical performance of the device. In addition, since it has gas barrier properties, the effect of extending the life of the device can be expected [1]. Poly-dichloro-para-xylylene (parylene-C) is a popular parylene derivative known for its low deposition temperature, transparency, and compatibility with standard nano- and micro-fabrication processes [2]. Parylene-C films are generally formed with the chemical vapor deposition (CVD) method introduced by William F. Gorham [3]. Generally, the CVD process proceeds in a vacuum and consists of four stages which are carried out in vaporization, pyrolysis, deposition, and trapping chambers, respectively. First, the parylene-C (powder) dimer is sublimed at a pre-programmed temperature range of 90–120 °C in the vaporization chamber. The sublimed dimer (di-para-xylylene) is then flowed through a heated pyrolysis chamber at 650 °C or more, causing the dimer to cleave into monomers (para-xylylene). The monomers are polymerized (into poly(para-xylylene)) directly on the surface of the substrates in the deposition chamber at room temperature. The combination of these vaporization and pyrolysis temperatures yields “transparent parylene” films on the substrate. The unpolymerized monomer gases are drained with a vacuum pump to the trapping chamber following their condensation with liquid nitrogen. In this way, the vacuum pump is protected against parylene contamination with residual monomers.

Much research on parylene has been conducted in micro-electromechanical systems (MEMS) [4], electro-mechanical sensors, the medical industry [5], and flexible electronic devices [6,7,8,9]. Owing to the fact that the transparent parylene film has a smooth surface, is flexible, and has high resistivity, it has been used as a substrate for flexible electronic devices and as an insulating layer for transistor devices [10,11]. It was also used as encapsulation and protective film [12,13,14,15,16] by leveraging its pinhole-free and moisture-resistant characteristics. In the preparation of parylene films through the CVD process, the quality of the parylene film is normally controlled by the working pressure of the deposition chamber, the temperature of the evaporation, and the pyrolysis chamber. Parylene-C films are normally deposited at a speed of approximately 30 nm/min. In a previous report, we demonstrated the preparation of parylene-C films with an ultrafast deposition speed (>500 nm/min) [6]. This was achieved by evaporating at temperatures more than the conventional temperature of 175 °C in the evaporation chamber, causing an increase in the dimer gas fraction and the deposition rate of parylene. Higher evaporation temperatures tend to enhance the deposition speed, causing insufficient pyrolysis, and as such, the polymerization process lowers the transmittance of deposited parylene films. A rapid increase in the evaporation chamber temperature to obtain abnormally fast deposition, however, prevents cracking of the vaporized parylene dimers in the cracking chamber, giving opaque parylene films with rough surfaces. To achieve sufficient pyrolysis of the dimer gas, the cracking chamber was modified with an internal structure followed by the pyrolysis of the dimers at 700 °C. This additional rod, which was located at the center of the pyrolysis chamber, guided the gas flow path through the hot wall of the pyrolysis chamber and ensured sufficient cleavage of the dimers. A thick transparent parylene film (applied as an OLED substrate) exhibiting transmittance and low haze was successfully deposited. Both slow- and fast-deposited parylene films were characterized and compared. It was demonstrated that the fast-deposited parylene films exhibited the same molecular structure and d-spacing as the slow-deposited parylene films. In another report, we investigated that pyrolysis of sublimed dimers (di-para-xylylene) at relatively low temperatures results in parylene-C films with light scattering properties (referred to as visible parylene films) due to the formation of uniformly distributed dimer crystals [9]. These films were applied as a light extraction layer for enhancing the outcoupling efficiency of bottom-emitting OLEDs. Even though previous studies have revealed the optical characteristics, such as the haze of the visible parylene films, a more comprehensive investigation of the structural, thermal, and electrical properties of these films is limited.

In this paper, the optical, electrical, and thermal characteristics of visible parylene films formed by tuning the pyrolysis temperature between 400 and 700 °C are analyzed. Fourier-transform infrared (FT-IR) spectrophotometry pointed to the formation of dimer crystals in the visible parylene films. In the X-ray diffractometry (XRD) analysis, the visible parylene films had large crystallite sizes of up to 1057.4 Å, compared to the transparent parylene films with crystallite sizes of approximately 90.2 Å. A relative reflectance of about 19% at 550 nm in the visible light region was achieved in the visible films. From the transmission line measurement (TLM), high resistivity of 2.8 × 10^10^ Ω cm was achieved in the visible parylene films. The single-step room temperature CVD fabrication of the visible parylene film as an insulation coating using commercially available parylene-C rendered it highly applicable in optoelectronic devices and optical systems.

## 2. Materials and Methods

**Figure 1:**
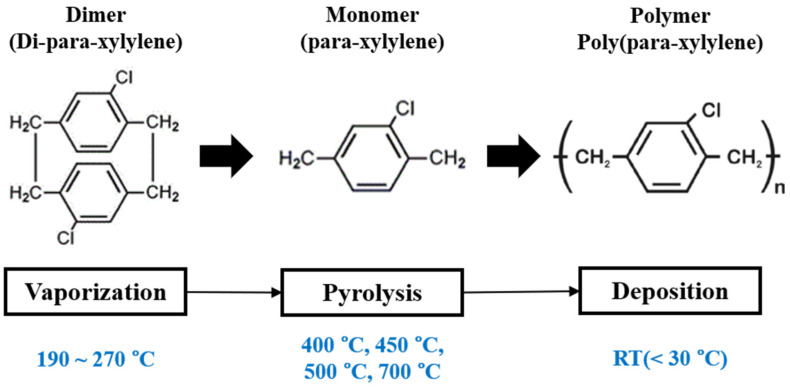
Molecular structure and schematic mechanism for the chemical vapor deposition process of visible parylene-C.

The visible parylene film was deposited using the parylene deposition system (EMBODY Tech, Daejeon, Korea). The molecular structure of parylene (dimer, monomer, and polymer) and the schematic mechanism for the chemical vapor deposition process of the visible parylene films are shown in Figure 1.

The film was obtained by vaporizing the parylene-C solid dimers at a maximum temperature of 270 °C in the vaporization chamber and then cleaving the dimer gases into monomers in the pyrolysis chamber. The pyrolysis of the dimer gases was controlled within the range of 400–700 °C in the pyrolysis chamber. The films produced by pyrolyzing at 400 °C, 450 °C, 500 °C, and 700 °C are denoted as P400, P450, P500, and P700, respectively. Before analyzing the optical, structural, and electrical properties of the parylene films, the bare glass substrates and the TLM pattern ITO substrates were cleansed via acetone sonication and dried in an oven for 15 min, respectively. The reflectance and transmittance spectra of the parylene films were analyzed within the 400–800 nm range using a UV-vis-NIR spectrophotometer (Lambda 950 UV-vis-NIR spectrophotometer, PerkinElmer, Waltham, MA, USA).

The haze was derived by determining the ratio of the measured diffuse transmittance to the total transmittance [9]. The thickness of the films was measured using an alpha step (Dektak-150, Veeco, Plainview, NY, USA). In addition, a scanning electron microscope (SEM, ZEISS Merlin Compact, Oberkochen, Germany) and an atomic force microscope (AFM, Shimadzu’s SPM-9600, Kyoto, Japan) were used for the surface analysis to confirm the crystallinity of the surface. The structural and thermal properties of the parylene films were analyzed using Fourier-transform infrared spectrophotometry (FT-IR, Nicolet 6700, Thermo Scientific, Waltham, MA, USA), X-ray diffraction (XRD, Rigaku SmartLab^®^, Tokyo, Japan), and differential scanning calorimetry (DSC, DSC1, Mettler Toledo), respectively. Finally, the electrical properties of the parylene films were measured through a probe station using a TLM pattern ITO substrate.

## 3. Results and Discussion

**Figure 2:**
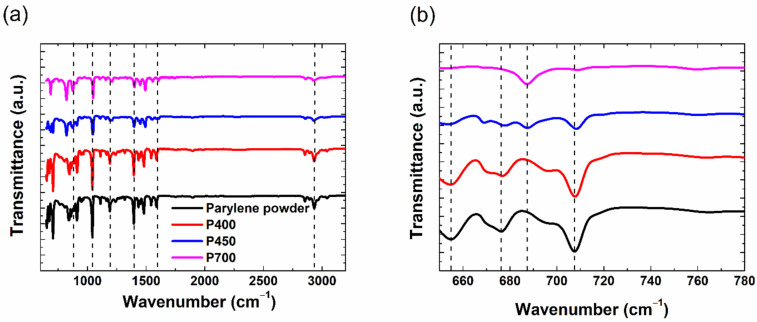
(**a**) FT-IR spectrum of the parylene powder, P400, P450, and P700 parylene films, (**b**) Enlarged FT-IR spectrum at 650–780 cm^−1^ of (**a**). The vertical dashed lines are for visual guidance only.

We performed FT-IR measurements to understand the structural characteristics of the parylene films when the pyrolysis temperature varied between 400 and 700 °C. In Figure 2a, the FT-IR spectrum of the parylene powder and the P400, P450, and P700 parylene films are compared.

Generally, in all spectra, the peaks corresponding to parylene-C appear at wavenumbers such as 3000, 1500, 1050, and 876 cm^−1^. The peaks at 2800–3000 cm^−1^ indicate that the absorption bands due to the symmetric and asymmetric stretching vibrations of sp3 and sp2 in C–H [17], while the peak at 1607 cm^−1^ indicates the skeletal aromatic C–C vibrations [18]. The peaks at 1500, 1450, and 815 cm^−1^ are due to the unsymmetrically tri-substituted benzene moiety, while the one at 1403 cm^−1^ is attributed to the C–C strain vibration, and that at 1198 cm^−1^ represents the in-plane modification of the C–H bond in the aromatic ring vibration [18]. The peak at 1050 cm^−1^ arises from the Cl–C bond of parylene [19]. Additionally, the peak at 876 cm^−1^ is attributed to a single H bound to the ring with the adjacent Cl and ethyl groups, while the peak representing an intramolecular C–H bond appears at 838 cm^−1^ [20]. The enlarged FT-IR spectrum at 650–780 cm^−1^ shows modified peak signals at 652, 675, and 709 cm^−1^, as shown in Figure 2b. The comparison with the FT-IR spectrum of the parylene powder confirmed that these peaks originated from the amount of parylene dimers. Thus, the peak signals at 652, 675, and 709 cm^−1^ could be attributed to the dimers, while that at 688 cm^−1^ is characteristic of the monomer. For instance, the ratio of the peak reduction for the dimer peak at 709 cm^−1^, relative to the parylene-C powder, is 0%, 63%, and 92% for the P400, P450, and P700 films, respectively. In our previous investigation, we demonstrated that higher evaporation temperatures (>175 °C) tend to enhance the deposition speed, but prevent the efficient cracking of the dimer crystals (in conventional pyrolysis chambers without additional features) [6]. The pyrolysis chamber used in this study was not modified to enhance efficient pyrolysis, even though the evaporation of the parylene powder was achieved at elevated temperatures (190~270 °C). Consequently, inefficient pyrolysis and the chance (approximately 10%) of depositing parylene dimers even at elevated pyrolysis temperatures (<500 °C) are inevitable. It was expected that the FT-IR characteristics of the P500 films were closely related to that of the P700 films. Thus, the parylene films pyrolyzed at low temperatures (<500 °C) have significant dimers in the films [6]. Therefore, the FT-IR analysis demonstrates that the cleavage rate of dimers to monomers is controlled by the pyrolysis temperature.

**Figure 3:**
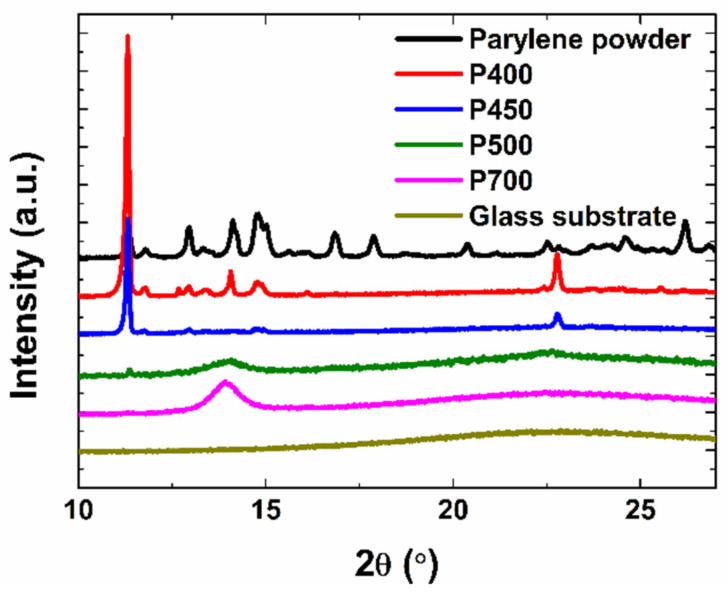
XRD patterns of the parylene powder; P400, P450, P500, and P700 parylene films; and the glass substrate.

Next, the crystal structure of the films was investigated through XRD measurement. XRD uses an X-ray diffraction image to obtain information about the components of a crystal structure, crystal size, and the state of poly-crystalline material. High and narrow peaks can be confirmed, as the film is produced with a low pyrolysis temperature, pointing to the fact that the sample is crystalline. Figure 3 shows the XRD patterns of the parylene powder and the P400, P450, P500, and P700 parylene films measured at 10–27°.

The peak at 2θ = 14.00° of the P700 and P500 parylene films corresponds to the (020) peak of the parylene polymer crystal [20,21]. Additionally, the parylene films pyrolyzed at low temperatures and the parylene powder in the dimer state show peaks at 2θ = 11.30°. The appearance of this peak could be attributed to the presence of dimers in the parylene films and not due to a new crystal bond generated from polymerization. These results are comparable with other reports in the literature. For instance, Z. Song et al. reported the structural characteristics of parylene-C dimers and parylene films [22]. For the thermally deposited film, the following steps were used: evaporation of the parylene-C dimers at 160 °C; pyrolysis at 650 °C; and deposition and polymerization of the parylene-C film at room temperature under a vacuum [22]. The XRD of the parylene-C dimer and parylene films include peaks at 2θ = 11.30° and 2θ = 14.00°, respectively. C. Chindam et al. also reported similar XRD patterns of parylene-C dimers [23]. Similar to the FT-IR analysis, there is a small number of dimer crystals present even in the films with dimer gases pyrolyzed at elevated temperatures (>500 °C). This could be attributed to the deposition of uncleaved dimer crystals in the film. Consequently, the XRD spectrum of the P500 film exhibits a small dimer peak attributed to the presence of a few dimer crystals. Bragg’s diffraction law (Equation (1)) can be used to calculate the d-spacing of the crystals, which represents the inter-planar spacing between the crystallite planes:

(1) $$d=\lambda /2\sin\theta_{B}$$

where d is the spacing, λ is the X-ray wavelength (1.5406 Å), and $\theta_{B}$ is the Bragg angle. The d-spacing of the P700, P500, P450, and P400 films were calculated as 6.3 Å, 6.3 Å, 7.8 Å, and 7.8 Å, respectively. Through this, it was found that although the pyrolysis temperature was different, the P700 and P500 films had the same d-spacing, as can also be noted for the P450 and P400 films. The crystallite size can be calculated according to Scherrer’s equation:

(2) $$D=K\lambda /B\cos\theta_{B}$$

where K is a dimensionless shape factor with a value of ~0.9, λ is the wavelength of the X-ray, B is the full width at half maximum (FWHM) of the diffraction peaks in radians, and $\theta_{B}$ is the Bragg angle. The crystallite sizes of the P700, P500, P450, and P400 films were calculated to be 90.2 Å, 67.8 Å, 851.1 Å, and 1057.4 Å, respectively. This difference is due to the proportion of uncleaved dimers in the P450 and P400 films. In contrast, in the P500 and P700 films, new crystals are formed by the polymer crystals generated during polymerization, and the higher the pyrolysis temperature, the larger the polymer crystals formed. However, it can be confirmed that the polymer crystals had relatively small crystallite sizes compared to the dimer crystals.

**Figure 4:**
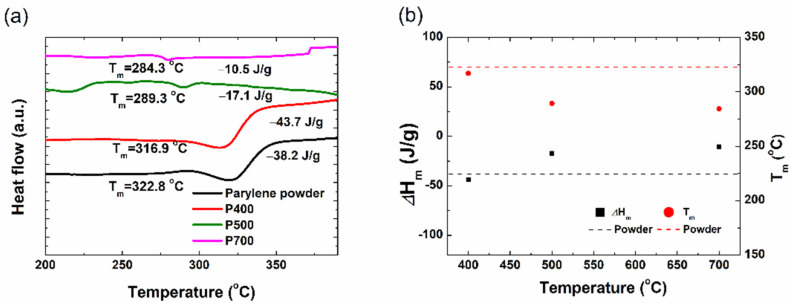
(**a**) DSC curves of the parylene powder; P400, P500, and P700 parylene films, (**b**) onset and peak melting temperatures.

To further understand how the difference in the crystal structure affected the change in the thermal properties of the parylene film, a DSC analysis was performed. In this method, the sample and reference materials are kept isothermal to each other while the heat flow as a function of temperature is recorded. The parylene-C films were cut into discs and subjected to DSC analysis at a rate of 10 °C/min. In Figure 4, the baseline-subtracted DSC curves of the parylene powder and the P400, P500, and P700 parylene films are compared.

The peak melting temperatures ($T_{m}$) are 284.3 °C, 289.3 °C, 317.0 °C, and 322.8 °C for the P700, P500, and P400 films and the parylene powder, respectively. The melting temperature of a polymer is generally affected by molecular weight, main chain stiffness, side group properties, and the interactions between the main chains [6]. The $T_{m}$ is a measure of the energy required to disrupt the crystal lattice [24]. Thus, the relatively high melting temperature of the visible parylene films compared to the P700 (transparent parylene) film can be attributed to the high number of dimer crystals, as confirmed in the XRD results. As shown in the XRD results, the P400 film and the parylene powder have relatively large crystal sizes and crystallinity. A high degree of crystallinity produces strong intermolecular attraction; thus, a high melting temperature is required. The high enthalpy of melting ($\Delta H_{m}$) of the film is closely related to the crystallinity of the film because more energy is required to melt the sample. The $\Delta H_{m}$ of the P700, P500, and P400 films are 10.48 J/g, 17.12 J/g, and 43.63 J/g, respectively. This indicates that the P400 films required high energy to melt due to the large crystallite size of the P400 dimer crystals. The P450 films were expected to exhibit slightly lower thermal characteristics ($T_{m},\;\mathrm{and}\;\Delta H_{m}$) than the P400 films since they also have a significant amount of dimer crystals, as revealed in the XRD results. Therefore, in the DSC analysis, more energy is required for melting the films.

**Figure 5:**
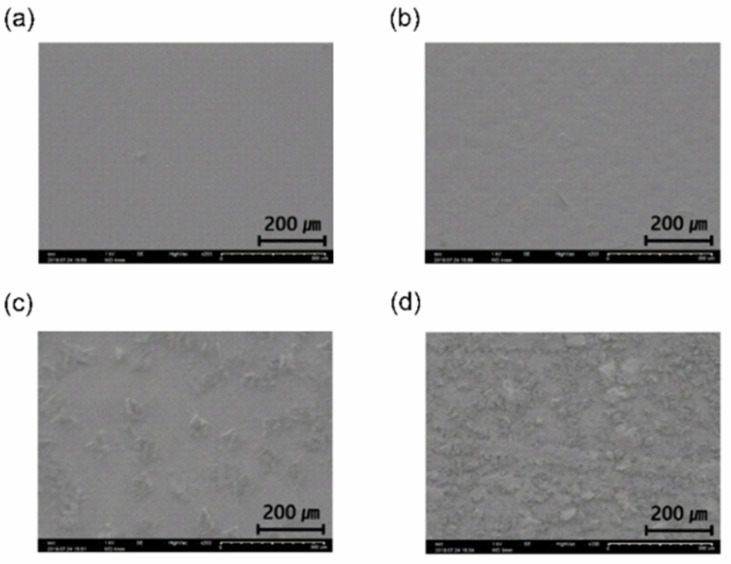
SEM images of the (**a**) P700 (**b**) P500 (**c**) P450 (**d**) P400 parylene films.

SEM measurement was performed to confirm the surface crystallinity of the parylene films. Figure 5 shows the surface properties of the P700, P500, P450, and P400 films deposited on a polyethylene naphthalate (PEN) film.

The P700 and P500 films are polymerized films with flat surfaces due to the high cleavage rate of the parylene dimers into monomers (a polymer). However, in the P450 and P400 films, randomly distributed dimer crystals can be seen due to the low dimer-cleavage rate. It was also demonstrated that the density of the dimer crystals on the surface of the P400 film was more than that of the P450 film.

**Figure 6:**
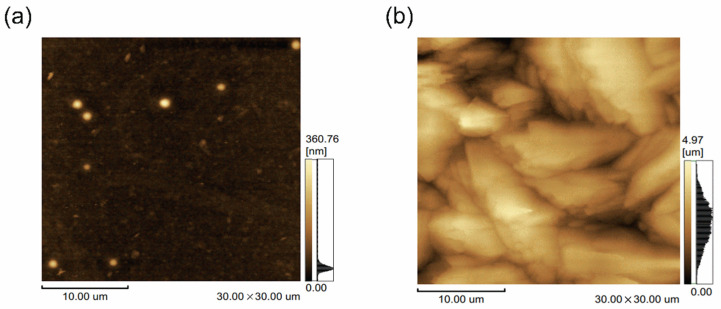
AFM images of (**a**) P700 (**b**) P400 parylene films.

The surface image of the 30 μm × 30 μm area of the parylene films is further analyzed through AFM images, as shown in Figure 6.

The surface roughness values ($R_{q}$) and peak-to-valley values of the P700 and P400 films are 17.6 nm, 332.1 nm, 817.7 nm, and 4440 nm, respectively, indicating a large difference in the surface roughness values. This can be attributed to the difference in crystallinity, as revealed in the previous SEM results. It was expected that the surface of the other visible films would also be several hundreds of nanometers rougher than the P700 film. A very small amount of crystal fragments is also observed in the P700 film, which can be attributed to some uncleaved dimer crystals since the same vaporization and pyrolysis chambers were used to produce the P400 film. However, these crystals were so small that they did not have a significant effect on the optical and physical properties of the P700 film.

**Figure 7:**
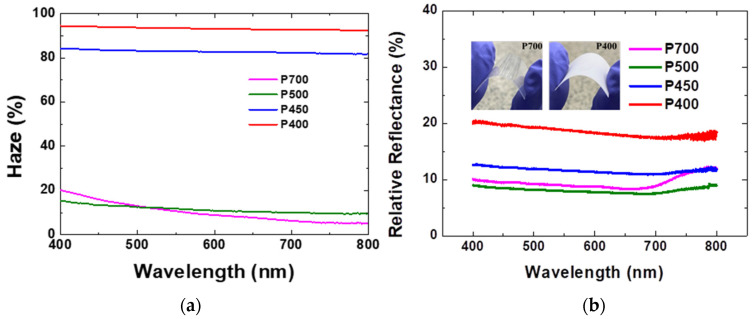
(**a**) The haze and (**b**) reflectance spectra of the P400, P450, P500, and P700 parylene films. Inset: photographs of the PET/P700 and PET/P400 films.

Pyrolysis at low temperatures in the range of 400–500 °C forms opaque parylene films due to the low dimer-cleavage rate. This can be confirmed through the transmittance and haze analysis of the visible parylene films. According to previously reported results, the P700, P500, P450, and P400 films showed a total transmittance of 80% or more at 550 nm [9]. In addition, haze, which is defined as the ratio of the diffuse transmittance to the total transmittance, was found to exceed 90% for P400 [9]. The haze of the parylene films are shown in Figure 7a. At 550 nm, the haze of the P700, P500, P450, and P400 parylene films are 10.2%, 12%, 85%, and 95%, respectively, a result which is consistent with the previous investigation [9]. The high haze of the visible parylene films could be attributed to the increased dimer crystals that enhanced the diffuse transmittance. Figure 7b shows the reflectance spectra of the P700, P500, P450, and P400 parylene films.

The relative reflectance measurement was performed to confirm the effect of the surface roughness of the parylene film on the optical properties. In the relative reflectance measurement, a barium sulfate (BaSO_4_) plate was used for a baseline measurement. The parylene films produced at low pyrolysis temperatures are visible because of the light scattering effect from the dimer crystals on the film surface. In addition, the reflectance increased because of the contribution of the light scattered in the direction opposite to the light propagation from the crystal fragment. The relative reflectance of the P700, P500, P450, and P400 films are found to be 8.6%, 7.9%, 11.6%, and 18.8%, respectively, at 550 nm in the visible light region. As shown with the SEM results, the P400 film has a significant amount of dimer crystals which could scatter the incident light. Consequently, the reflectance of the P400 film is the highest compared to the other visible parylene films obtained at relatively higher pyrolysis temperatures.

**Figure 8:**
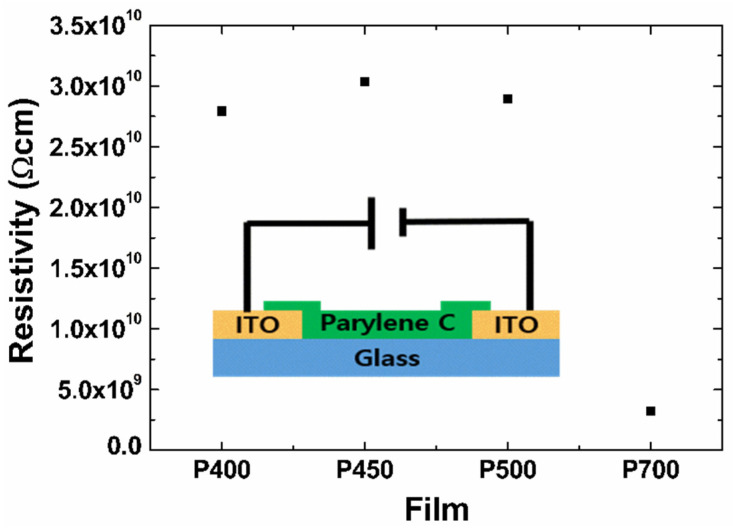
The specific resistance of the P700, P500, P450, and P400 parylene films. Inset: schematic structure of the device and the measurement setup.

The TLM analysis was performed to understand the insulation properties of the parylene films. TLM is a method of calculating contact resistance using horizontal electrodes [25,26]. Figure 8 shows the resistivity of the P700, P500, P450, and P400 parylene films calculated through the TLM.

The resistivity, ρ, is extracted from the calculated conductivity by measuring the semi-conductor contact resistance using the channel lengths of 50, 75, 100, 150, and 200 μm, respectively. This can be derived from the following equation:

(3) $$\rho =WT\left(R_{total}-2R_{c}\right)/L$$

where W is the channel width, T is the film thickness, $R_{total}$ is the total resistance, $R_{c}$ is the contact resistance generated at the interface between the metal and the parylene film, and L is the channel length. The thicknesses of the P700, P500, P450, and P400 films are 1.3, 3.4, 6.1, and 3.4 μm, respectively. The resistivities of the P700, P500, P450, and P400 films, calculated using Equation (3), are 3.2 × 10^9^ Ω cm, 2.9 × 10^10^ Ω cm, 3.0 × 10^10^ Ω cm, and 2.8 × 10^10^ Ω cm, respectively. The low resistivity of the P700 film can be attributed to the low electrical resistance in the film due to the small crystallite size demonstrated in the XRD analysis of Figure 3. All parylene films have relatively superior insulation performance due to their high resistance. However, the films formed at low pyrolysis temperatures have a disadvantage in that the amount of polymer composite is small, and the film is mechanically weak. Therefore, it is proposed that an optimized dimer-to-polymer ratio is ensured for the visible films to achieve the desired mechanical strength and electrical insulation.

Y.-C. Tai et al. reported parylene-pyrolyzed carbon for micro-electro-mechanical systems (MEMs) applications [27]. One μm-thick parylene-C films were pyrolyzed at different temperatures in an N_2_ atmosphere up to 900 °C. The resistivity was calculated from the measured sheet resistance and film thickness. Parylene-C films pyrolyzed at low pyrolysis temperatures exhibited high resistivity. The resistivity became less than 1.0 × 10^10^ Ω cm above 600 °C and decreased to approximately 1.0 × 10^−2^ Ω cm at 900 °C [27]. It is worth noting that in their report, the pyrolysis of the thin parylene film involved the deposition of the parylene film on substrates, followed by pyrolysis by heating in the absence of O_2_. Consequently, extra experimental setups and time are required to tune the resistivity of the parylene films. However, in our study, we have demonstrated that by controlling the pyrolysis of the dimers (di-para-xylylene) in the CVD process, the resistivity of the parylene films can be easily tuned without additional processes. In other reports, such as the one from R. J. Mammone et al., the dielectric/resistivity properties of parylene-C films were modified with ion implantation [28]. For instance, the measured resistivities through the film fell from 2.4 × 10^11^ Ω cm for unirradiated films to approximately 6.9 × 10^6^ Ω cm for films irradiated with 10^16^ Cl^+^ ions/cm^2^ and to 2.1 × 10^6^ Ω cm for films irradiated with 10^16^ Ar^+^ ions/cm^2^ [28]. In our study, the resistivity of the visible parylene films could be easily controlled by tuning the pyrolysis of the parylene dimers. This investigation of the optical and electrical characteristics of visible parylene films is expected to find wide applications in visible insulation film coating technology for major semi-conductor devices and advance electronic devices at large.

## 4. Conclusions

In this study, the effect of pyrolysis temperature on the structural, thermal, optical, and electrical properties of parylene films was investigated. High haze and transmittance could be achieved by controlling the pyrolysis temperatures for the parylene dimers. Through FT-IR and XRD measurements, it was confirmed that a visible parylene film was formed due to the presence of dimer crystals in the film. The enthalpy of melting of the film with the dimer crystals was greater than 40 J/g, whereas that of the polymer melts was near 15 J/g. Through optical measurements, it was demonstrated that the surface roughness caused by the dimer crystals affects the reflectance of the parylene films, resulting in opaque films. In addition, it was demonstrated that visible parylene films could achieve insulation performance with a resistivity of 3.0 × 10^10^ Ω cm. This single-step room temperature CVD fabrication process, using commercially available parylene-C, is expected to be utilized as visible insulation coatings for various electronic devices.

## Data Availability

The data presented in this study are available upon request from the corresponding author.
